# Effects of hyperprotein diet on anxiety, haemodynamics and morphofunctional aspects of the heart of Wistar rats

**DOI:** 10.1113/EP090638

**Published:** 2023-03-29

**Authors:** Flander Diego de Souza, Thiago Montes Fidale, Talita Cristina Rodrigues Pereira, Matheus Matioli Mantovani, Simone Ramos Deconte, Daniel Moreira‐Silva, Francyelle Borges Rosa de Moura, Letícia de Queiroz Martins, Luciano Alex dos Santos, Robson da Silva Medeiros, Marcos Luiz Ferreira Neto, Elmiro Santos Resende

**Affiliations:** ^1^ Experimental Medicine Laboratory Federal University of Uberlândia‐UFU Uberlândia MG Brazil; ^2^ Institute of Biotechnology / Department of Medicine Federal University of Catalão Catalão GO Brazil; ^3^ Federal University of Uberlandia Uberlândia MG Brazil; ^4^ Institute of Biomedical Sciences / Department of Physiology Federal University of Uberlândia Uberlândia MG Brazil; ^5^ Federal University of ABC, UFABC São Paulo SP Brazil; ^6^ Federal University of Minas Gerais Belo Horizonte MG Brazil; ^7^ Federal University of Uberlândia Uberlândia MG Brazil; ^8^ Department of Physiological Sciences Federal University of Uberlandia‐UFU Uberlândia MG Brazil; ^9^ Faculty of Medicine / Posgraduate Program in Health Sciences Federal University of Uberlandia Uberlândia MG Brazil; ^10^ Institute of Biotechnology / Biology Institute Federal University of Catalão‐Goiás Catalão GO Brazil

**Keywords:** anxiety, heart, high protein diet

## Abstract

Anxiety is a mechanism preparatory to a response in situations of threat and danger, involving behavioural, affective and physiological factors. Protein‐based foods have a high concentration of amino acids which perform multiple functions, including in the biosynthesis of excitatory transmitters for the central nervous system. In recent years, adherence to high‐protein diets has been gaining ground in society, on the basis that it brings benefits to the musculoskeletal system and cardiovascular health. The aim of the present study was to investigate the effect of a high‐protein diet in a state of anxiety and to investigate morphofunctional cardiovascular effects of a high‐protein diet in Wistar rats. The experiment lasted 8 weeks and two groups of male rats were submitted to either a normoproteic or a hyperproteic diet. Anxiety was assessed using the plus maze test and cardiovascular morphofunctional aspects using transthoracic echocardiography and invasive measurements of femoral blood pressure. There was no statistically significant difference in the anxiety test, but the hyperproteic group was more agitated, with greater displacement during the test. Changes were found in systolic and end‐diastolic volume, left ventricular diameter in systole and heart rate, which were significantly lower in the hyperproteic group, and there was an increase in the thickness of the interventricular septum in diastole. The results showed no influence of the higher protein diet on the animals’ anxiety, body weight and haemodynamics.

## Abstract (highlights)

- **What is the central question of this study?**
  The consumption of a high‐protein diet has been associated with an anxiogenic factor that can influence anxiety and possible cardiovascular changes: does the consumption of a high‐protein diet interfere with anxiety, haemodynamics and morphofunctional aspects of the heart of Wistar rats?
- **What is the main finding and its importance?**
  Our study showed that the high‐protein diet did not interfere with anxiety and haemodynamics. The animals in the hyperproteic group showed positive heart adaptations characterized by less work and lower heart rate without impairing ejection fraction and systemic blood pressure.

## 1 INTRODUCTION

Anxiety is listed in the scale of psychological disorders affecting approximately 7.3% of the world's population (Craske et al., 2016). It is a preparatory mechanism put into effect in situations of threat and danger, involving behavioural, cognitive, physiological and affective factors (Cardozo et al., 2016). Anxious individuals believe that the probability of negative events occurring is very high, feel threatened by danger and may react in the wrong way, becoming hostage to their obsessive thoughts (Moura et al., 2018). When there is this potential danger of anxiety, neural connections are signalled by glutamate to retain information and make it easier to learn about the related danger (Carobrez, 2003).

Protein‐based foods contain a high concentration of glutamate (Carobrez, 2003), the major excitatory neurotransmitter of the central nervous system and responsible for 75% of the electrical activity in the brain (Tortora & Grabowski, 2002). In recent years, adherence to high‐protein diets has been gaining ground in society (Aparicio et al., 2013). This has been an effective strategy in controlling and reducing body weight, especially in obese individuals (Bantle et al., 2008; Lepe et al., 2011), stimulating people's satiety and, consequently, reducing food intake (Peseta & Samuel, 2014).

Studies show that a high‐protein diet is beneficial for the cardiovascular system, justifying the interest in understanding this influence on haemodynamic responses (Pavlou et al., 2018; Sabaka et al., 2017). Although the benefits of a high‐protein diet on health and body composition are already well established, the effect of this food strategy on anxiety, haemodynamics and morphofunctional aspects of the heart still need to be elucidated. The aim of this study was to evaluate the effect of a high‐protein diet versus a normoproteic diet on these variables, in an experimental model using Wistar rats.

## 2 METHODS

### 2.1 Ethics approval

The experiments took place at Federal University of Uberlândia's Rodent Vivariums's Network (REBIR‐UFU) and all the procedures were in accordance with the ethics protocol of the Brazilian College of Animal Experimentation (COBEA). The study was approved by the local Committee on Ethics for the Animals utilization (CEUA), with favourable opinion number 179/18.

### 2.2 Animals and diet

Twenty‐nine male Wistar rats were used, with an average age of 3 months and body weight between 250 and 300 g, supplied by the REBIR‐UFU. The animals were divided into two groups, one the control group (C) with 14 animals fed a standard normoproteic diet, the other (H) with 15 animals fed with a 40% protein diet. The control group was provided with a standard (Nuvilab CR‐1 diet, Quimtia Brasil, Chapecó ‐ SC ‐ CEP: 89.804.13), with the nutritional composition shown in the Table 1. The composition of this diet reached the nutritional requirements for adult rats, according to the American Institute of Nutrition recommendations (AIN 93M).

**TABLE 1:** Macronutrient composition of commercial feed Nuvilab CR1 (Nuvital) of the normoproteic group.

Nutrient	Diet (100 g)
Carbohydrate	57
Protein	22
Lipid	4
Ash	9
Humidity	8
Gross energy (kcal/g)	3.5

The rats were housed in collective boxes with a maximum capacity of five animals per box, and had free access to food and water. Monitoring boxes and water bottles were exchanged for sterilized material twice a week, according to the REBIR protocol. Food was replaced at the same frequency and leftovers were calculated and discarded. Body weight was monitored weekly using a precision scale. The duration of the experiment was about 8 weeks and during this period only one animal from the control group was lost.

The handling process, the cleaning of the cages and the food and water re‐position were performed in a biosafety cabinet with BS60 BioSafety cabinet airflow (Tecniplast, Milan, Italy). The environment was controlled in terms of ventilation, temperature (21°C) and humidity ranging from 40 to 60%. Throughout the research, a 12‐h light–dark cycle (light from 07.00 to 17.00 h) was established.

The commercial feed was adapted for the high‐protein group to meet the nutritional requirements of an adult rat. According to the American Institute of Nutrition recommendations (AIN 93M), high‐protein diets have to contain 25–40% crude protein. To reach the required concentration, an additional 18% of protein was added to the control group's diet. For this purpose, the product ‘Whey Protein’ Isolated, Provon 292 from (Glanbia Nutritionals, Monções, São Paulo/SP, CEP 04.571‐000) was used giving a hyperproteic diet with 40% crude protein (Table 2).

**TABLE 2:** Macronutrient composition table of the hyperproteic feed with the addition of Whey Protein Isolate to the standard feed Nuvilab CR1 (Nuvital).

Nutrients	Diet (100 g)
Carbohydrate	57.7
Protein	40
Lipid	4.16
Gross energy (kcal/g)	4.2

### 2.3 Anxiety assessment

**FIGURE 1:**
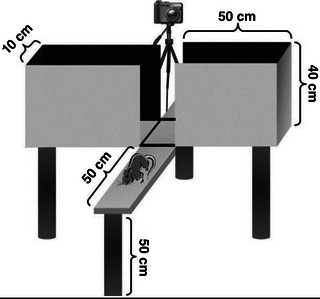
Eleated plus maze test apparatus. Source: Sweis et al. (2016).

For the assessment of anxiety, the elevated plus maze test (LCE) was applied, which is an instrument used to measure anxiety in rats (Carobrez & Bertoglio, 2005). The equipment consists of two open arms and two closed arms, each one measuring 50 × 10 cm, being crossed by arms of the same size forming a cross. The equipment details are shown in Figure 1.

The closed arms have 40 cm‐high side walls, except in the middle, delimiting a central platform of 10 × 10 cm. The environment was moderately lit and each animal was placed towards one of the open arms. In this position, the animal was left for 5 min to make free exploration of the environments. The animal's entrance into the environment was defined as being when it placed all four paws on the arms, and each session was recorded with the aid of a digital camera. After each test, the platform was sanitized with 70% ethanol (Barzegar et al., 2015; Roohbakhsh et al., 2007).

The parameters measured in the maze were expressed as the time the animal remained in the open arms and the total number of entries. A high frequency in which the open side is explored represents low anxiety and the total number of entries represents the animal's motor activity.

### 2.4 Transthoracic echocardiography

Structural and functional analysis of the heart was performed by transthoracic echocardiography on eight randomly selected animals from each group. All animals were anesthetized with 0.1 ml/100 g of ketamine (10%) with the same dose of xylazine (2%). An anterior thorax trichotomy was performed on the animals and they were placed in a 45° lateral decubitus position. We used a GE Healthcare (Milwaukee, WI, USA) ultrasound device, model Logiq‐F6, and an 8.0 MHz transducer with a depth of 3.0 cm and a sector angle of 75°. All the procedures were performed at the university's veterinary hospital. The measurements of end‐diastolic diameter (LVEDD) and left ventricular end‐systolic diameter (LVSD) were performed using M‐mode and the right parasternal transversal view, in the plane of the tendinous cords. These measurements were used to calculate the shortening fractional (FS) and left ventricular ejection fraction (LVEF) by the method of Teichholz et al. (1976).

### 2.5 Invasive recording of blood pressure and heart rate

Six rats from each group were randomly selected for invasive haemodynamic analysis. The surgical procedure was performed in the last week of the study, and the animals were preliminarily anaesthetized with sodium thiopental (40 mg/kg, i.v.); afterwards, the animals inhaled halothane (2%) mixed with 100% oxygen (O_2_). Cannulation of the femoral artery was performed to register blood pressure, using a polyethylene catheter filled with liquid and connected to a pressure transducer (mechanical–electrical) coupled to a BP100 amplifier (ADInstruments, Colorado Springs, CO, USA) and the Power Lab/8‐channel record scanning system (ADInstruments). Data acquisition was performed using registration system software (LabChart v 8.0). To analyse heart rate variability over time and frequency domains, the CardioSeries program was used (Penteado, 2012).

### 2.6 Histological analysis of the left ventricle

Morphological alterations were measured by quantifying the area of the left ventricular wall and the diameter of the left ventricular lumen (Ventura et al., 2015). For these evaluations, hearts were fixed for 3 h in Metacarn (methanol, acetic acid and chloroform in a ratio of 6:3:1). Then, the samples were embedded in paraffin and sectioned at 5 μm with a rotating microtome (Microm HM‐315, Bunker Lake Blvd. Ramsey, USA) at the level of the ventricles. The sections were stained with haematoxylin and eosin and then scanned (Aperio AT, Leica Microsystems, Wetzlar, Germany).

The area of the left ventricle (cardiac muscle) was obtained by subtracting the area of the left ventricle lumen from the value obtained for the total area of this ventricle. In addition, we used the lumen area to obtain the left ventricular lumen diameter, using the following formula: diameter = 2×√ area/π.

### 2.7 Animal killing and blood collection

After 8 weeks of experiment, the animals were anaesthetized using 0.1 ml/100 g of 10% ketamine (Syntec do Brasil Ltda, Santana de Parnaíba, SP, Brazil), associated with the same dose of 2% xylazine (Rhobifarma Indústria Farmacêutica Ltda., Hortolândia, SP, Brazil), and an abdominal aorta puncture was performed to obtain blood products causing death by exsanguination. All procedures took place on the premises of REBIR‐UFU.

### 2.8 Statistical analysis

The results were expressed as means ± standard deviation (SD). The normality of the sample distribution was tested using the Shapiro–Wilk test. For normal data, comparisons were performed by analysis of variance (ANOVA), followed by Tukey's test, when necessary, or by Student's *t*‐test. The statistical analysis was performed using the GraphPad Prism statistical package (version 5.03, GraphPad Software, San Diego, CA, USA). The statistical significance was established at *P* ≤ 0.05.

## 3 RESULTS

### 3.1 Feed consumption and animal weight

The mean weekly feed intake was similar in both groups. There was no statistically significant difference in the body weight of the animals between the control group and the group supplemented with high‐protein chow (Table 3).

**TABLE 3:** Weekly feed consumption and animal weight.

Variable	Control (*n* = 14)	Hyperproteic (*n* = 15)	Δ%	*P*
Feed consumption (g)	738.9 ± 43.8	690.4 ± 26.5	6.56%	0.359
Body weight (g)	371.3 ± 12.3	384.2 ± 11.1	3.47%	0.448

Results expressed as mean ± SD. Δ%: percentage change.

### 3.2 Elevated plus maze test

Table 4 presents the mean result of the elevated plus maze (EPM) test referring to the length of stay in the open arms (in seconds) and the total number of entries.

**TABLE 4:** Eleated plus maze test.

	Time in open arms (s)		Total number of entries	
Group	4 weeks	8 weeks	4 weeks	8 weeks
Control (*n* = 14)	22.0 ± 18.9	27.1 ± 34.5	2.3 ± 1.57	1.8 ± 1.66
Hyperproteic (*n* = 15)	24.0 ± 32.2	12.5 ± 15.2	2.5 ± 2.06	4.1 ± 2.71

Results expressed as means ± SD.

A statistical analysis was performed at the two points on the timeline in which the test was performed. There were no intragroup differences for the control (*P* = 0.889) or the hyperproteic (*P* = 0.523) group. In the intergroup analysis, no difference was found in the fourth (*P* = 0.948) and eighth week (*P* = 0.433).

**FIGURE 2:**
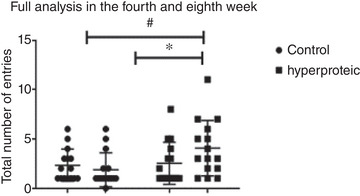
Analysis of the total number of entries in the EPM test in different situations: fourth and eighth week. Number of animals: Control (14) and Hyperproteic (15). Results expressed as means ± SD. #Intergroup difference, *intragroup difference, *P* ≤ 0.05.

For statistical analysis of the number of total entries in the EPM test, the results were analysed at the fourth and eighth weeks. There was a significant difference in the intragroup comparison only in the group that received a high‐protein diet (*P* ≤ 0.020), which had the highest number of entries. In the intergroup analysis, this same occurrence was only seen at the eighth week (*P* ≤ 0.012) (Figure 2).

### 3.3 Invasive haemodynamic analysis and heart rate and blood pressure variability

**FIGURE 3:**
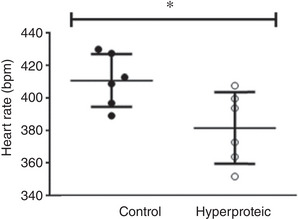
Analysis of heart rate variability performed in the last week of the experiment. Results expressed as means ± SD. *P ≤ 0.05.

The invasive haemodynamic study showed that the animals in the high‐protein diet group had a lower heart rate; there was no statistical difference in blood pressure between the groups (Table 5 and Figure 3).

**TABLE 5:** Haemodynamic analysis.

Variable	Control (*n* = 6)	Hyperproteic (*n* = 6)	*P*
MAP (mmHg)	125.2 ± 7.5	121.6 ± 4.6	0.347
SBP (mmHg)	146.2 ± 8.5	147.8 ± 5.8	0.702
DBP (mmHg)	107.9 ± 8.3	102.0 ± 4.6	0.157
HR (bpm)	410.7 ± 16.2	381.4 ± 22.1	0.025*

Results expressed as means ± SD. *P value ≤ 0.05. Abbreviations: DBP, diastolic blood pressure; HR, heart rate; MAP, mean arterial pressure; SBP, systolic blood pressure.

The analysis of heart rate variability showed no statistically significant differences in the time domain for the mean of the cardiac interval (CI) or for the SD of the RR intervals, or for the root‐mean‐square of the differences between normal R‐R intervals (RMSSD) (Table 6).

**TABLE 6:** Heart rate variability in the time domain.

Variable	Control (*n* = 5)	Hyperproteic (*n* = 5)	*P*
CI	149.2 ± 3.1	158.4 ± 9.9	0.081
SD	5.8 ± 1.0	7.4 ± 1.9	0.142
Variance	34.6 ± 11.8	58.2 ± 29.6	0.135
RMSSD	4.0 ± 0.2	4.2 ± 0.8	0.455

Results expressed as means ± SD. Abbreviations: CI, mean cardiac interval; RMSSD, mean square root of the differences between normal R intervals; SD, SD of RR intervals; Variance, variance of RR intervals.

The analysis of the cardiac interval in the frequency domain showed no significant differences between the groups for the mean CI, for the absolute and null low (LF) and high (HF) frequency components, for the LF/HF ratio (Table 7).

**TABLE 7:** Heart rate variability in the frequency domain.

Variable	Control (*n* = 5)	Hyperproteic (*n* = 5)	*P*
CI	149.6 ± 3.4	158.8 ± 9.7	0.081
LF ABS	1.7 ± 0.9	3.3 ± 2.5	0.212
HF ABS	4.1 ± 0.6	5.4 ± 2.1	0.236
LF nu	27.4 ± 11.1	32.6 ± 13.7	0.528
HF nu	72.6 ± 11.1	67.4 ± 13.7	0.528
LF/HF	0.420 ± 0.233	0.584 ± 0.348	0.406

Results expressed as means ± SD. LF and HF absolute (ABS) and null (null) low (LF) and high frequency (HF) components. CI, mean cardiac interval frequency domain.

The analysis of blood pressure variability in the time domain showed no differences in mean (mmHg), SD and variance. In the frequency domain, there were no differences in relation to the mean (ms) and to the LF absolute component, and no significant alterations in baroreflex sensitivity baroreflex effectiveness index (BEI) (Table 8).

**TABLE 8:** Variability of blood pressure in the time domain, frequency and baroreflex sensitivity.

Time domain variable	Control (*n* = 5)	Hyperproteic (*n* = 5)	*P*
Mean (mmHg)	143.8 ± 8.0	149.0 ± 7.6	0.325
SD	4.8 ± 0.9	5.5 ± 0.6	0.215
Variance	24.0 ± 9.1	30.3 ± 6.5	0.238
Domain frequency			
Average (ms)	143.8 ± 8.0	149.0 ± 7.6	0.325
LF ABS	9.6 ± 2.5	9.0 ± 2.8	0.755
Baroreflex sensitivity			
BEI ALL	0.086 ± 0.018	0.120 ± 0.041	0.128

Results expressed as mean ± SD. SD, standard deviation of RR intervals; Variance, variance of RR intervals; LF (ABS), absolute low frequency component; BEI ALL, Baroreflex sensitivity.

### 3.4 Transthoracic echocardiography

**FIGURE 4:**
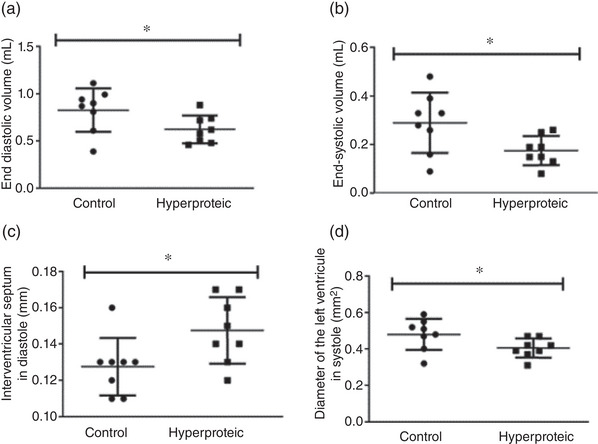
Echocardiogram analysis with significant differences between groups. Results expressed as means ± SD. *P ≤ 0.05.

In the echocardiographic analysis, it was observed that the LV end‐systolic volume was lower in the high‐protein diet group and that the thickness of the interventricular septum in the systole was greater in this same group. There was also a trend towards an increase in LV end‐diastolic volume (*P* = 0.052), reduction of left ventricular diameter in systole (*P* = 0.054) and diastole (*P* = 0.065). The results are shown in the Table 9 and Figure 4.

**TABLE 9:** Echocardiographic analysis of the heart.

Variable	Control (*n* = 8)	Hyperproteic (*n* = 8)	*P*
FE (%)	65.87 ± 8.03	72.00 ± 5.45	0.117
FS (%)	32.12 ± 5.57	36.5 ± 4.76	0.137
VDF (ml)	0.82 ± 0.21	0.62 ± 0.13	0.05*
VSF (ml)	0.29 ± 0.115	0.17 ± 0.05	0.033*
SIVd (mm)	0.12 ± 0.014	0.14 ± 0.017	0.034*
SIVs (mm)	0.20 ± 0.041	0.22 ± 0.026	0.394
DIVEs	0.48 ± 0.08	0.40 ± 0.049	0.05*
DIVEd	0.71 ± 0.074	0.64 ± 0.049	0.065

Results expressed as means ± SD. *P ≤ 0.05. DIVEDd, Left ventricular diameter at diastole; DIVEs, Left ventricular diameter in systole; FE, ejection fraction; FS, systolic fraction; HR, heart rate; SIVd, Interventricular septum in the diastole; SIVs, Interventricular septum in the systole; VDF, End diastolic volume; VSF, End systolic volume.

### 3.5 Assessment of the left ventricular area

**FIGURE 5:**
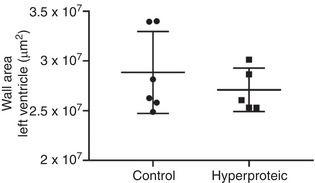
Area occupied by the cardiac muscle in the left ventricular wall.

**FIGURE 6:**
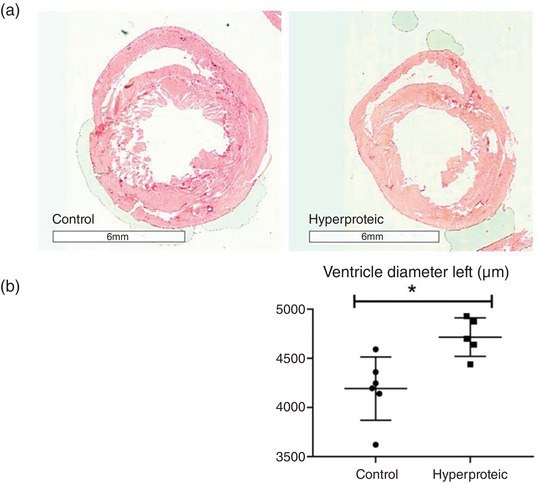
Left ventricular lumen diameter. Results expressed as means ± SD. *P ≤ 0.05. (A) photomicrographs stained with haematoxylin and eosin and (B) graph demonstration of the quantification of ventricular diameter. The hyperproteic group had higher mean values (12.5%) for the diameter of the left ventricular lumen when compared to the control, *P ≤ 0.05.

There was no statistical difference in the area of the left ventricular wall between the groups evaluated. The results are shown in Figures 5 and 6.

## 4 DISCUSSION

The relationship between the consumption of a high‐protein diet and anxiety and morphofunctional aspects of the heart are not well understood yet, and this was analysed in a pioneering way in the present study.

It has already been described that diets rich in protein (with 25% of the total energy as protein) induce greater weight loss when compared to a diet rich in carbohydrates (Skov et al., 1999). In the present study, a group of Wistar rats received a diet with approximately 40% protein and the results were compared with a control group with a normoproteic diet. At the end of 8 weeks, no statistically significant difference was found for the total body weight of the animals. Farnsworth et al. (2003) showed that reducing the total calorie consumption is more important for weight reduction than a high protein content. However, in a study analysing the diet of healthy American volunteers, it was found that individuals who consumed protein in amounts that exceeded the daily dietary recommendations had lower body mass index (Pasiakos et al., 2015).

Although the comparison with other studies that did not use the same experimental model with rats is inappropriate, one of the possibilities for the non‐occurrence of body weight reduction in animals that received a high‐protein diet in the present study is the distribution of macronutrients in the diet. Another explanation may be related to the relatively short time of exposure to a high‐protein diet. Both issues should be investigated in future experiments with other diet formulations.

A study using a high‐protein diet offered ad libitum for a period of 12 weeks resulted in greater satiety than a high‐protein diet offered in an isocaloric manner (Weigle et al., 2005). It is noteworthy that protein is the macronutrient with the greatest thermic effect on the digestive and absorptive process (Leidy et al., 2015), and high protein content in the diet contributes to weight reduction due to the increase of the basal metabolic rate (Halton & Hu, 2004). The absence of weight reduction at the end of the present study could be linked to the higher caloric density in the group that consumed the high‐protein diet.

Protein provides the body with essential and non‐essential amino acids that are used in the biosynthesis of new proteins and neurotransmitters. High protein‐based intake can affect brain functioning and mental health by producing different amounts of neurotransmitters (Sathyanayana et al., 2008). The hypothesis tested in the present study is that the high‐protein diet would exert an anxiogenic effect due to the amino acid profile of the diet. Unlike the initial hypothesis of our study, Haghighatdoost et al. (2016) when evaluating the influence of glycaemic rate on anxiety and depression suggested that reducing refined carbohydrates and including more protein may be beneficial for mental health. In a case report of a patient with generalized anxiety disorder, it was found that changes in macronutrient composition, reducing carbohydrates, especially refined ones, and adding more protein and lipids to the diet, could have a beneficial effect on decreasing of clinical symptoms of anxiety (Aucoin & Bhardwaj, 2016).

In the elevated plus maze test it is possible to verify the animal's state of anxiety based on the exploration of the open arm performed by the animal over the investigation time. In this aspect of the test, no difference was found between the groups tested related to behaviour. Another variable evaluated in this same test was the total number of entries of the animal. This result is associated with the motor activity and it was higher in week 8 in the high‐protein diet group, for both comparisons, intragroup and intergroup.

Studies have shown that anxiety related to psychosocial stress is associated with a higher risk of cardiovascular mortality (Emdin et al., 2016; Rosengren et al., 2004) and one of the focuses of the present research was to try to understand the relationship of the high‐protein diet to cardiovascular health from the analysis of the haemodynamic behaviour of the animals. Previous studies have shown that a high‐protein diet associated with low carbohydrate intake produces a reduction in blood pressure (Dong et al., 2013; Te Morenga et al., 2011). Evaluating the protective effect of the high‐protein diet (40%) in three study groups, control, whey protein and casein, Singh et al. (2016) found a reduction in systolic blood pressure, especially in the group that received whey.

The amount of crude protein offered in the hyperproteic group of the present study was 40%. In an animal model, Martin et al. (2015) evidenced attenuation of the increase in systolic blood pressure associated with age in animals that consumed a diet with a high amount of whey protein, one of the reasons this haemodynamic response could be related to the amount of dipeptides capable of inhibiting angiotensin converting enzyme. Other studies have shown that changes in dietary protein consumption affect the behaviour of systolic blood pressure in animals and also in humans (Engen & Swenson, 1969). In the present study, using haemodynamic invasive measurements, there were no differences in systolic, diastolic and mean blood pressure, showing that in the proposed model, the high‐protein diet did not produce the changes already described in the literature. It should be noted that the hypotensive effect has not been a consistent finding in the literature, and a meta‐analysis that investigated the use of a high‐protein diet compared with other types of diets in patients with type 2 diabetes mellitus did not demonstrate significant differences in blood pressure (Yu et al., 2020).

When analysing parasympathetic and sympathetic modulation and baroreflex sensitivity, we did not find any significant difference. Despite the fact that the hyperproteic group presented a higher rate of the low frequency component, which is derived from the joint action of the vagal and sympathetic components on the heart, the results did not reach statistical significance. Oliveira et al. (2004) showed that a low‐protein diet promotes an increase in the baseline mean blood pressure, heart rate and R‐R variability. Our study showed that the high‐protein group had a significant reduction in the heart rate in comparison to the control group.

The echocardiographic evaluation showed that the end‐systolic and end‐diastolic volume, and the diameter of the left ventricle in systole were significantly smaller in the group that received the high‐protein diet, and the interventricular septum thickness in diastole was also greater in the animals that received the high‐protein diet. These results suggest a model of greater contraction efficiency for the hyperproteic group, with less work demanded from the maintenance of cardiac function. The histological analyses showed a greater area of ventricular lumen in the hyperproteic group, suggesting a cardiac remodelling for unknown reasons.

These differences were not followed by changes in left ventricular ejection fraction, which keeps both groups within physiological values. This study is the first to our knowledge designed to analyse the effect of a high‐protein diet on anxiety, heart rate variability, heart morphology and also systolic blood pressure.

## 5 CONCLUSION

We conclude that the high‐protein diet does not interfere in the animal's anxiety, from the evidence from the elevated plus maze test. The analysis of the R‐R variability does not show alterations for the modulation of the autonomic nervous system that could be related to the results found.

It is also concluded that the animals submitted to the high‐protein diet showed positive adaptations in the heart characterized by less work, lower heart rate and without damage to the ejection fraction and to systemic blood pressure.

## AUTHOR CONTRIBUTIONS

Experiments were carried out at the Network of Rodent Vivariums of the Federal University of Uberlândia (REBIR‐UFU). In addition to the UFU Veterinary Hospital for echocardiographic analysis and the UFU Institute of Biomedical Sciences (ICBIM‐UFU) for the preparation of histological slides. Flander Diego de Souza, Thiago Montes Fidale, Marcos Luiz Ferreira Neto, Elmiro Santos Resende: conception and design of the work; acquisition and analysis of data interpretation for work; essay writing and critical review of important intellectual content. Talita Cristina Rodrigues Pereira: data collection during the survey; analysis of data interpretation for work. Matheus Matioli Mantovani: responsible for the echocardiographic examination of the animals; analysis of data interpretation for work. Simone Ramos Deconte: preparation of slides and histological analysis; critical review of important intellectual content. Daniel Moreira Silva: elevated plus maze statistics; critical review of important intellectual content. Francyelle Borges Rosa de Moura: histological slides statistics; critical review of important intellectual content. Letícia de Queiroz Martins, Robson da Silva Medeiros: analysis of data interpretation for the work. Luciano Alex dos Santos: feed preparation and nutritional calculations. All authors have read and approved the final version of this manuscript and agree to be accountable for all aspects of the work in ensuring that questions related to the accuracy or integrity of any part of the work are appropriately investigated and resolved. All persons designated as authors qualify for authorship, and all those who qualify for authorship are listed.

## CONFLICT OF INTEREST

The authors report no conflicts of interest.

## FUNDING INFORMATION

No funding was received for this work.

## Supporting information

Statistical Summary Document

## Data Availability

Data supporting the findings of this study are available from the corresponding author upon reasonable request.
