# Supplementary material for: Effects of hyperprotein diet on anxiety, haemodynamics and morphofunctional aspects of the heart of Wistar rats
Source: Exp Physiol. 2023 Mar 29;108(6):818–26. doi: 10.1113/EP090638 (PMC10988522; doi:10.1113/EP090638)
Supplement: Supplementary file 1 — Statistical Summary Document [file EPH-108-818-s001.docx]

**Manuscript Title:** Effects of Hyperprotein Diet on Anxiety, Hemodynamics and Morphofunctional Aspects of the Heart of Wistar Rats

**Authors:** Flander Diego de Souza, Thiago Montes Fidale, Talita Cristina Rodrigues Pereira, Matheus Matioli Mantovani, Simone Ramos Deconte, Daniel Moreira Silva, Francyelle Borges Rosa de Moura, Letícia de Queiroz Martins, Luciano Alex dos Santos, Robson da Silva Medeiros, Marcos Luiz Ferreira Neto, Elmiro Santos Resende.

**Animal model used, if applicable:** Wistar Isogenic Rat (Rattus norvegicus)

**Underlying hypothesis:** This investigation tests the hypothesis that the consumption of a high-protein diet can alter anxiety behavior and promote morphofunctional changes in the heart.

**Definitions of ‘n’:**

| 1. Weekly feed consumption and animal weight |
| --- |
| 2. Protein-rich diet and anxiety |
| 3. Hemodynamic behavior and heart rate |
| 4. Difference between functional and structural capacity of the heart of animals submitted to a high protein diet |
| 5. Structural alteration of the heart of animals histologically |

**Statistical summary table:**

| **Experimental question number** | **Finding/ conclusion** | **Experimental location/ variable** | **Mean value** | **SD** | **n** | **P**** | **Units** | **Data comparisons** | **Statistical test** | **Any other variable** | **Figure/ table in which data are presented** | **Comments**  **e.g. observation** |
| --- | --- | --- | --- | --- | --- | --- | --- | --- | --- | --- | --- | --- |
| 1. Difference in weekly feed intake and weight of animals in each group | There was no difference between feed intake and body weight of the animals. | Body weight | Body weight: 371,3g | 12,3 | 29 | 0,448 | n/D | Control group x Hyperproteic group | t tests (and nonparametric tests) | --- | Table 3 | no observation |
|  | There was no difference between feed intake and body weight of the animals. | Feed offered | Feed consumption: 738,9g | 43,8 | 29 | 0,359 | n/D | Control group x Hyperproteic group | t tests (and nonparametric tests) | --- | Table 3 | no observation |
| 2. Protein-rich diet and anxiety | There was no difference in anxiety behavior between the groups. | Eleated Plus Maze (EPM) Test | Time in open arms (seconds)  4 weeks (Control group 22,0) (H Hyperproteic group: 24,0)  8 weeks (Control group: 27,1) (Hyperproteic group: 12,5) | Time in open arms (seconds)  4 weeks (Control group: 18,9) (Hyperproteic group: 34,5)  8 weeks (Control group: 32,2) (Hyperproteic group: 15,2) | 29 | --- | n/D | Control group x Hyperproteic group | Two-way ANOVA | --- | Table 4 | Comments  e.g. observation |
| 3. Hemodynamic behavior and heart rate | There was no difference in hemodynamics and cardiac variability. However, the heart rate between groups showed a difference. | Hemodynamic and heart rate analysis by femoral cannulation | FE (%):Control group: 65,87 and Hyperproteic group: 72,00 | FE (%):Control group: 8,03 and Hyperproteic group: 5,45 | 12 | **0,025** | n/D | Control group x Hyperproteic group | t tests (and nonparametric tests) | --- | Table 5 | For hemodynamic analysis, the animals were randomized to femoral cannulation. The number of animals was enough to generate statistically valid results. |
|  |  |  | MAP (mmHg): Control group: 125,2 and Hyperproteic group: 121,6 | MAP (mmHg): Control group: 7,5 and Hyperproteic group: 4,6 | 12 | 0,347 | n/D | Control group x Hyperproteic group | t tests (and nonparametric tests) | --- | Table 5 |  |
|  |  |  | SBP (mmHg): Control group: 146,2 and Hyperproteic group: 147,8 | SBP (mmHg): Control group: 8,5 and Hyperproteic group: 5,8 | 12 | 0,702 | n/D | Control group x Hyperproteic group | t tests (and nonparametric tests) | --- | Table 5 |  |
|  |  |  | DBP (mmHg): Control group: 107,9 and Hyperproteic group: 102,0 | DBP (mmHg): Control group: 8,3 and Hyperproteic group: 4,6 | 12 | 0,157 | n/D | Control group x Hyperproteic group | t tests (and nonparametric tests) | --- | Table 5 |  |
|  |  |  | HR (bpm): Control group: 410,7 and Hyperproteic group: 16,2 | HR (bpm): Control group: 381,4 and Hyperproteic group: 22,1 | 12 | 0,025 | n/D | Control group x Hyperproteic group | t tests (and nonparametric tests) | --- | Table 5 |  |
|  |  | Hemodynamic and heart rate analysis by femoral cannulation.Heart rate variability in the time domain | CI: Control group: 149,2 and Hyperproteic group: 158,4 | CI: Control group: 3,1 and Hyperproteic group: 9,9 | 10 | 0,081 | n/D | Control group x Hyperproteic group | t tests (and nonparametric tests) | --- | Table 6 |  |
|  |  |  | SD: Control group: 5,8 and Hyperproteic group: 7,4 | SD: Control group: 1,0 and Hyperproteic group: 1,9 | 10 | 0,142 | n/D | Control group x Hyperproteic group | t tests (and nonparametric tests) | --- | Table 6 |  |
|  |  |  | VARIANCE: Control group: 34,6 and Hyperproteic group: 58,2 | VARIANCE: Control group: 11,8 and Hyperproteic group: 29,6 | 10 | 0,135 | n/D | Control group x Hyperproteic group | t tests (and nonparametric tests) | --- | Table 6 |  |
|  |  |  | RMSSD: Control group: 4,0 and Hyperproteic group: 4,2 | RMSSD: Control group: 0,2 and Hyperproteic group: 0,8 | 10 | 0,455 | n/D | Control group x Hyperproteic group | t tests (and nonparametric tests) | --- | Table 6 |  |
|  |  | Hemodynamic and heart rate analysis by femoral cannulation. Heart rate variability in the frequency domain | CI: Control group: 149,6 and Hyperproteic group: 158,8 | CI: Control group: 3,4 and Hyperproteic group: 9,7 | 10 | 0,081 | n/D | Control group x Hyperproteic group | t tests (and nonparametric tests) | --- | Table 7 |  |
|  |  |  | LF ABS: Control group: 1,7 and Hyperproteic group: 3,3 | LF ABS: Control group: 0,9 and Hyperproteic group: 2,5 | 10 | 0,212 | n/D | Control group x Hyperproteic group | t tests (and nonparametric tests) | --- | Table 7 |  |
|  |  |  | HF ABS: Control group: 4,1 and Hyperproteic group: 5,4 | HF ABS: Control group: 0,6 and Hyperproteic group: 2,1 | 10 | 0,236 | n/D | Control group x Hyperproteic group | t tests (and nonparametric tests) | --- | Table 7 |  |
|  |  |  | LF nu: Control group: 27,4 and Hyperproteic group: 32,6 | LF nu: Control group: 11,1 and Hyperproteic group: 13,7 | 10 | 0,528 | n/D | Control group x Hyperproteic group | t tests (and nonparametric tests) | --- | Table 7 |  |
|  |  |  | HF nu: Control group: 72,6 and Hyperproteic group: 67,4 | HF nu: Control group: 11,1 and Hyperproteic group: 13,7 | 10 | 0,528 | n/D | Control group x Hyperproteic group | t tests (and nonparametric tests) | --- | Table 7 |  |
|  |  |  | LF/HF: Control group: 0,420 and Hyperproteic group: 0,584 | LF/HF: Control group: 0,233 and Hyperproteic group: 0,348 | 10 | 0,406 | n/D | Control group x Hyperproteic group | t tests (and nonparametric tests) | --- | Table 7 |  |
|  |  | Hemodynamic and heart rate analysis by femoral cannulation. Variability of blood pressure in the time domain, frequency and baroreflex sensitivity.  Time domain variables. | Average (mmHg): Control group: 143,8 and Hyperproteic group: 149,0 | Average (mmHg): Control group: 8,0 and Hyperproteic group: 7,6 | 10 | 0,325 | n/D | Control group x Hyperproteic group | t tests (and nonparametric tests) | --- | Table 8 |  |
|  |  |  | SD: Control group: 4,8 and Hyperproteic group: 5,5 | SD: Control group: 0,9 and Hyperproteic group: 0,6 | 10 | 0,215 | n/D | Control group x Hyperproteic group | t tests (and nonparametric tests) | --- | Table 8 |  |
|  |  |  | VARIANCE: Control group: 24,0 and Hyperproteic group: 30,3 | VARIANCE: Control group: 9,1 and Hyperproteic group: 6,5 | 10 | 0,238 | n/D | Control group x Hyperproteic group | t tests (and nonparametric tests) | --- | Table 8 |  |
|  |  | Hemodynamic and heart rate analysis by femoral cannulation. Variability of blood pressure in the time domain, frequency and baroreflex sensitivity.  Domain Frequency | Average (ms): Control group: 143,8 and Hyperproteic group: 149,0 | Average (ms): Control group: 8,0and Hyperproteic group: 7,6 | 10 | 0,325 | n/D | Control group x Hyperproteic group | t tests (and nonparametric tests) | --- | Table 8 |  |
|  |  |  | LF ABS: Control group: 9,6 and Hyperproteic group: 9,0 | LF ABS: Control group: 2,5 and Hyperproteic group: 2,8 | 10 | 0,755 | n/D | Control group x Hyperproteic group | t tests (and nonparametric tests) | --- | Table 8 |  |
|  |  | Hemodynamic and heart rate analysis by femoral cannulation. Variability of blood pressure in the time domain, frequency and baroreflex sensitivity.  Baroreflex Sensitivity. | BEI ALL: Control group: 0,086 and Hyperproteic group: 0,120 | BEI ALL: Control group: 0,018 and Hyperproteic group: 0,041 | 10 | 0,128 | n/D | Control group x Hyperproteic group | t tests (and nonparametric tests) | --- | Table 8 |  |
| 4. Difference between functional and structural capacity of the heart of animals submitted to a high protein diet | We obtained significant differences in relation to cardiac function between the animals | Transthoracic Echocardiography | FE (%): Control group: 65,87 and Hyperproteic group: 72,00 | FE (%):Control group: 8,03 and Hyperproteic group: 5,45 | 16 | 0,117 | n/D | Control group x Hyperproteic group | t tests (and nonparametric tests) | --- | Table 9 | The animals were randomized because the same animals submitted to the femoral cannulation procedure could not participate in the echocardiographic analysis. The number of animals undergoing echocardiography met the requirements to generate statistically valid data. |
|  |  |  | FS (%):Control group: 32,12 and Hyperproteic group: 36,5 | FS (%):Control group: 5,57 and Hyperproteic group: 4,76 | 16 | 0,137 | n/D | Control group x Hyperproteic group | t tests (and nonparametric tests) | --- | Table 9 |  |
|  |  |  | VDF (ml): Control group: 0,82 and Hyperproteic group: 0,62 | VDF (ml): Control group: 0,21 and Hyperproteic group: 0,13 | 16 | **0,05** | n/D | Control group x Hyperproteic group | t tests (and nonparametric tests) | --- | Table 9 |  |
|  |  |  | VSF (ml): Control group: 0,29 and Hyperproteic group: 0,17 | VSF (ml): Control group: 0,115 and Hyperproteic group: 0,05 | 16 | **0,033** | n/D | Control group x Hyperproteic group | t tests (and nonparametric tests) | --- | Table 9 |  |
|  |  |  | SIVd (mm): Control group: 0,12 and Hyperproteic group: 0,14 | SIVd (mm): Control group: 0,014 and Hyperproteic group: 0,017 | 16 | **0,034** | n/D | Control group x Hyperproteic group | t tests (and nonparametric tests) | --- | Table 9 |  |
|  |  |  | SIVs (mm): Control group: 0,20 and Hyperproteic group: 0,22 | SIVs (mm): Control group: 0,041 and Hyperproteic group: 0,026 | 16 | 0,394 | n/D | Control group x Hyperproteic group | t tests (and nonparametric tests) | --- | Table 9 |  |
|  |  |  | DIVEs: Control group: 0,48 and Hyperproteic group: 0,40 | DIVEs: Control group: 0,08 and Hyperproteic group: 0,049 | 16 | **0,05** | n/D | Control group x Hyperproteic group | t tests (and nonparametric tests) | --- | Table 9 |  |
|  |  |  | DIVEd: Control group: 0,71 and Hyperproteic group: 0,64 | DIVEd: Control group: 0,074 and Hyperproteic group: 0,049 | 16 | 0,065 | n/D | Control group x Hyperproteic group | t tests (and nonparametric tests) | --- | Table 9 |  |
| 5. Structural alteration of the heart of animals histologically | No change in left ventricular area, but difference in left ventricular lumen diameter | Histological analysis of the heart | Left ventricle diameter: Control group: 3763 and Hyperproteic group: 4716,2 | Left ventricle diameter: Control group: 1175,1 and Hyperproteic group: 195,6 | 12 | **0,011** | n/D | Control group x Hyperproteic group | t tests (and nonparametric tests) | --- | Figura 6 | We had difficulties to make the cardiac slides of all the animals. However, the number of animals presented in the table was sufficient for statistical analysis. |
|  |  |  | Left ventricular wall area: Control group: 29577325,0 and Hyperproteic group: 27096995,8 | Left ventricular wall area: Control group: 4228527,8 and Hyperproteic group: 2181169,1 | 12 | 0,417 | n/D | Control group x Hyperproteic group | t tests (and nonparametric tests) | --- | Figura 5 |  |
